# The Role of the Interleukin-17 Axis and Neutrophils in the Pathogenesis of Endemic and Systemic Mycoses

**DOI:** 10.3389/fcimb.2020.595301

**Published:** 2020-12-14

**Authors:** Juan David Puerta-Arias, Susana P. Mejía, Ángel González

**Affiliations:** ^1^Medical and Experimental Mycology Group, Corporación para Investigaciones Biológicas (CIB), Universidad de Antioquia, Medellín, Colombia; ^2^School of Health Sciences, Universidad Pontificia Bolivariana, Medellín, Colombia; ^3^Max Planck Tandem Group in Nanobioengineering, Universidad de Antioquia, Medellin, Colombia; ^4^Basic and Applied Microbiology Research Group (MICROBA), School of Microbiology, Universidad de Antioquia, Medellin, Colombia

**Keywords:** IL-17, Neutrophils, *Paracoccidioides* spp., *Coccidioides* spp., *Histoplasma capsulatum*, *Blastomyces* spp

## Abstract

Systemic and endemic mycoses are considered life-threatening respiratory diseases which are caused by a group of dimorphic fungal pathogens belonging to the genera *Histoplasma*, *Coccidioides*, *Blastomyces*, *Paracoccidioides*, *Talaromyces*, and the newly described pathogen *Emergomyces*. T-cell mediated immunity, mainly T helper (Th)1 and Th17 responses, are essential for protection against these dimorphic fungi; thus, IL-17 production is associated with neutrophil and macrophage recruitment at the site of infection accompanied by chemokines and proinflammatory cytokines production, a mechanism that is mediated by some pattern recognition receptors (PRRs), including Dectin-1, Dectine-2, TLRs, Mannose receptor (MR), Galectin-3 and NLPR3, and the adaptor molecules caspase adaptor recruitment domain family member 9 (Card9), and myeloid differentiation factor 88 (MyD88). However, these PRRs play distinctly different roles for each pathogen. Furthermore, neutrophils have been confirmed as a source of IL-17, and different neutrophil subsets and neutrophil extracellular traps (NETs) have also been described as participating in the inflammatory process in these fungal infections. However, both the Th17/IL-17 axis and neutrophils appear to play different roles, being beneficial mediating fungal controls or detrimental promoting disease pathologies depending on the fungal agent. This review will focus on highlighting the role of the IL-17 axis and neutrophils in the main endemic and systemic mycoses: histoplasmosis, coccidioidomycosis, blastomycosis, and paracoccidioidomycosis.

## Introduction

Systemic fungal infections are characterized by their ability to produce a potentially life-threatening respiratory disease. These systemic mycoses are caused by a group of thermally dimorphic fungal pathogens belonging to different genera of several species including *Histoplasma capsulatum*, *Coccidioides* spp., *Blastomyces* spp., *Paracoccidioides* spp., *Talaromyces marneffei* and the newly described pathogen *Emergomyces* spp. (Sepúlveda et al., 2017; Turissini et al., 2017; Kirkland and Fierer, 2018; Cao et al., 2019; Schwartz et al., 2019; Schwartz and Kauffman, 2020). Additionally, these mycoses are usually geographically restricted; thus, histoplasmosis is found worldwide, coccidioidomycosis is endemic in some regions of the United States and some countries of Latin America, blastomycosis is endemic in North America and Africa, paracoccidioidomycosis is restricted to Latin America, talaromycosis is endemic in Asian countries, while emergomycosis has been reported in Africa, Europe, Asia, and North America (Sepúlveda et al., 2017; Turissini et al., 2017; Kirkland and Fierer, 2018; Cao et al., 2019; Schwartz et al., 2019; Schwartz and Kauffman, 2020).

In general, these systemic mycoses are acquired by inhalation of the conidia or spores that are produced in the mold phase; in the lungs, a temperature-dependent transformation occurs to the yeast phase, except for *Coccidioides* spp., which undergoes isotropic growth to form spherules initials (Hung et al., 2007). These fungal morphotypes are phagocytized by macrophages and can spread hematogenously to various organs, causing disseminated infection; nonetheless, the clinical presentation could vary from self-limited, or mild, to severe infection, which, in turn, depends on several factors including the immune response and the inoculum size, among others.

Several studies have confirmed the T-cell mediated immune response to some of these dimorphic fungal pathogens, especially those associated with T helper (Th)1 and Th17 responses that are essential for protection (Wüthrich et al., 2011; Nanjappa et al., 2012; Wu et al., 2013; Ketelut-Carneiro et al., 2019). Of note, Th17 and IL-17 protective responses, which also participate during the primary infections in the nonimmune host, are associated with recruiting and activating neutrophils and macrophages to the site of infection as well as with chemokine and proinflammatory cytokines production, a mechanism mediated by the fungal recognition of pattern recognition receptors (PRRs) present on the surface of the host cells, which lead to the activation of adaptor molecules and the subsequent downstream signaling (Wüthrich et al., 2011; Nanjappa et al., 2012; Wu et al., 2013; Ketelut-Carneiro et al., 2019). Nonetheless, both the Th17/IL-17 axis and neutrophils appear to play a dual role, being beneficial mediating fungal controls and detrimentally promoting disease pathology depending on the fungal agent (Loures et al., 2009; Wüthrich et al., 2011; Pino-Tamayo et al., 2016; Puerta-Arias et al., 2016; Ketelut-Carneiro et al., 2019).

In this review, we will discuss the current findings regarding the role of the IL-17 axis and neutrophils in the immune response against dimorphic fungal pathogens with special emphasis on the most studied endemic and systemic mycoses: histoplasmosis, coccidioidomycosis, blastomycosis, and paracoccidioidomycosis. Of note, the role of IL-17 and neutrophils on talaromycosis and emergomycosis have not been investigated so far or are incipient, reasons why these mycoses were not included in this review.

## IL-17: Sources and Function

The IL-17 family is a group of pleiotropic cytokines secreted mainly by a subset of CD4+T helper cells (Th) known as Th17 cells (Harrington et al., 2006). The differentiation and stimulation of Th17 from naïve CD4+ T-cells occurs in secondary lymphoid organs with the participation of IL-1β, IL-6, transforming growth factor β (TGFβ), and IL-23. The stimuli with cytokines trigger the downstream STAT3, promoting the activation of *RORγt*, the master transcriptional factor, which modulates the production of the hallmark cytokines IL-17A, IL-17F, and other cytokines such as IL-21, IL-22, and granulocyte-macrophage colony-stimulation factor (GM-CSF) (Hartupee et al., 2009; Isailovic et al., 2015; Monin and Gaffen, 2018). Moreover, polarized Th17 cells express CC chemokine receptor 6 (CCR6), which allows their migration into mucosal barrier sites (Abusleme and Moutsopoulos, 2017).

Moreover, other cell populations were also reported as important sources of IL-17A and IL-17F, such as the CD8+ T-cells and the innate immune cells including γδ T-cells (Takatori et al., 2008), innate lymphoid cells subset 3 (ILC3) (Geremia et al., 2011; Villanova et al., 2014), invariant natural killer cells (iNKT) (Michel et al., 2007), IL-17 innate lymphoid cells (ILC17) (Buonocore et al., 2010), and natural killer T (NKT) cells (Cella et al., 2009). Additionally, macrophages and dendritic cells are also important sources of IL-23 and IL-17, which are produced in response to the microorganism’s invasion and inflammatory cytokines stimulation. Neutrophils and mast cells also contribute to IL-17 production (Hoshino et al., 2008; Lin et al., 2011; Monin and Gaffen, 2018; Schön and Erpenbeck, 2018).

The IL-17 family includes six related proteins, namely IL-17A, IL-17B, IL-17C, IL-17D, IL-17E (also known as IL-25), and IL-17F (Monin and Gaffen, 2018), IL-17A (commonly known as IL-17) being the most studied member of the IL-17 family. The functions of IL-17 are crucial to maintaining mucosal immunity against extracellular and intracellular pathogens through the induction of antimicrobial proteins and the recruitment of neutrophils to the site of infections. Furthermore, IL-17 increases mucosal barrier repair and maintenance by the production of tight junction proteins and the stimulation of epithelial cell proliferation (Valeri and Raffatellu, 2016).

IL-17 is recognized by the family of the IL-17 receptors (IL-17R), which is a multimeric receptor constituted by two subunits with five members: IL-17A-IL17E. The IL-17R is composed of a common IL-17RA chain and a second chain that determines the ligand and downstream signal. (Yao et al., 1995; Toy et al., 2006; Rickel et al., 2008; Ramirez-Carrozzi et al., 2011; Zhu et al., 2011). The IL-17RA is expressed on the surface of leukocytes, keratinocytes, fibroblasts, epithelial, mesothelial, and vascular endothelial cells. The expression of IL-17RA induces granulopoiesis, neutrophil recruitment, and inflammatory response (Tristão et al., 2017). The IL-17A and IL-17F share a high degree of similarity, with both playing a central role in the adaptive immune response, especially against bacteria and fungi. IL-17A and IL-17F induce an inflammatory response by stimulating the expression of proinflammatory cytokines and chemokines and matrix metalloproteinase (MMP) production, thus promoting a potent immune response with the recruitment of immune cells to the site of infection, mainly neutrophil accumulation (Tristão et al., 2017). IL-17A induces the production of the chemokines CXCL1, CXCL2, and CXCL8 (IL-8), which in turn attract neutrophils. In addition, IL17A appears to have a protecting role against microorganisms through the induction of antimicrobial peptides, including β-defensins, S100A8, and lipocalin 2 (Onishi and Gaffen, 2010; Chen and Kolls, 2017).

Fungal infections have been particularly associated with the regulation of the Th17 immune response by the activation of CD4+ T - antigen-presenting cells *via* recognition of the components of the fungal cell wall by pattern recognition receptors (PRRs). The cell walls of fungal pathogens contain three major polysaccharides types: β-glucan, chitin, and mannan (Netea et al., 2008); meanwhile, PRRs include Dectin-1, Dectin-2, Dectin-3, Mincle, mannose receptor (MR), and *Toll-*like receptors (TLRs), among others; thus, Dectin-1 recognizes fungi *via* β-1,3-glucan. Dectin-2 and Mincle recognize mannose-like structures, while TLR2 recognizes mainly β-glucan and zymosan, and TLR4 recognizes mannan components (McGreal et al., 2006; Netea et al., 2006; Reid et al., 2009; Yamasaki et al., 2009; Saijo et al., 2010; Ishikawa et al., 2013). Once PRRs recognize fungal cells, these interactions trigger a cascade of signaling events, with the participation of cytosolic adaptors [mainly caspase adaptor recruitment domain family member 9 (Card9) and myeloid differentiation factor 88 (MyD88)] that transduce signals from these PRRs, that in turn activate the secretion of proinflammatory cytokines and the induction of T-cell differentiation (Drummond et al., 2011; Loures et al., 2011; Wüthrich et al., 2012).

On the whole, the activation of the Th17 immune response against fungal infection depends upon which receptors are involved and the degree of interaction (Netea et al., 2008; van de Veerdonk et al., 2009; Wang et al., 2016).

## The Role of IL-17 Axis in The Dimorphic Fungal Infections

It is known that the development of Th1 cells is crucial for protective immunity against dimorphic fungal pathogens including *H. capsulatum*, *Coccidioides* spp., *Blastomyces* spp., and *Paracoccidioides* spp.; however, the roles of the Th17 cell and IL-17 are controversial. In models of infection with the above fungal pathogens, some studies have shown that Th17/IL-17 axis mediate resistance, while others have shown that they promote disease pathology (Deepe and Gibbons, 2009; Loures et al., 2009; Loures et al., 2009; Wüthrich et al., 2011; Nanjappa et al., 2012; Wu et al., 2013; Wang et al., 2014; Pino-Tamayo et al., 2016; Puerta-Arias et al., 2016; Ketelut-Carneiro et al., 2019). In the case of histoplasmosis, coccidioidomycosis, and blastomycosis, it has been reported that mice vaccinated against these three fungal pathogens showed that Th1 immunity was dispensable, whereas the fungal-specific Th17 cells were sufficient for inducing protection against these systemic mycoses (Wüthrich et al., 2011). Subsequently, these results were confirmed using hosts lacking CD4+ cells, where CD8+ T-cell derived IL-17 was indispensable to develop immunity protection against these three endemic and systemic mycoses (Nanjappa et al., 2012).

**Figure 1:**
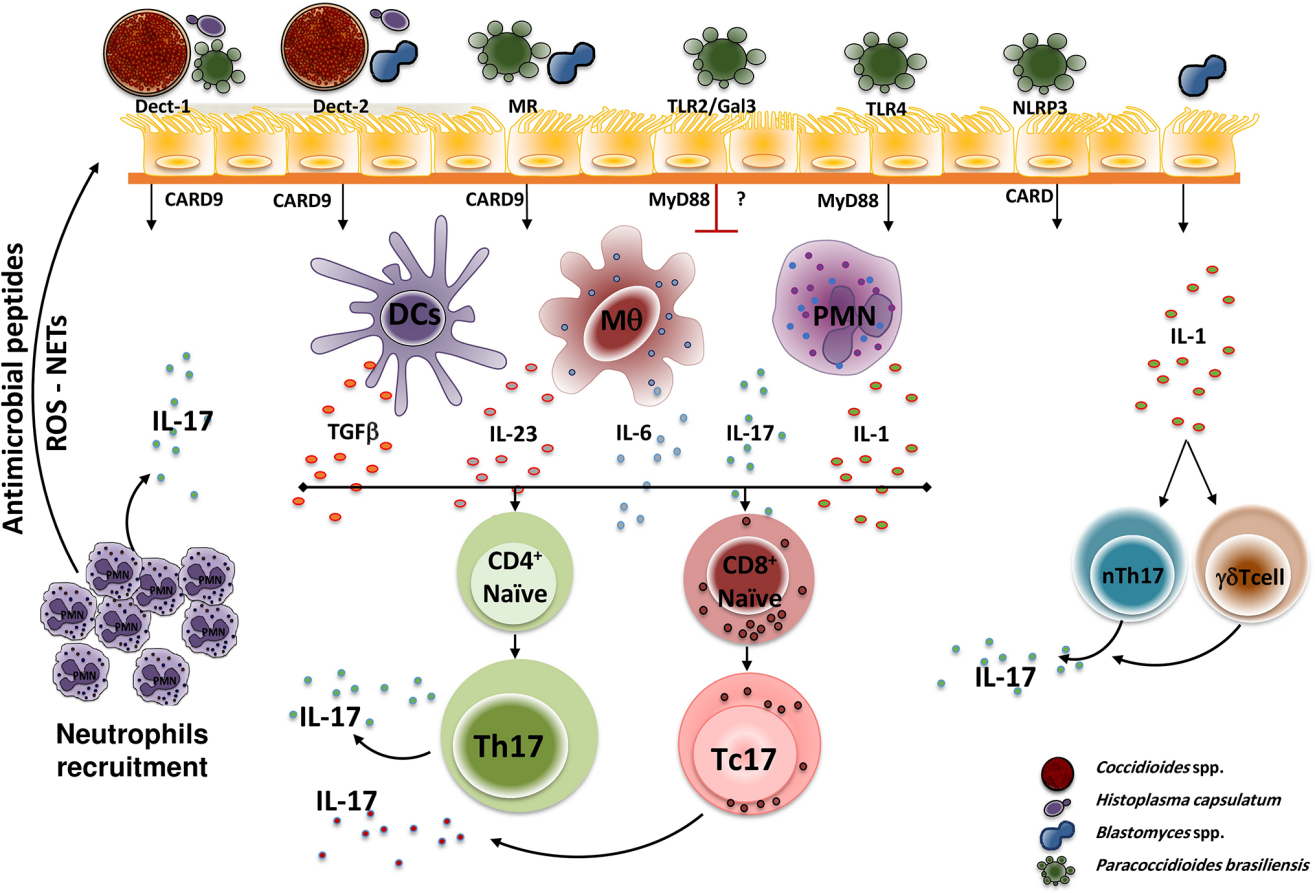
Dimorphic fungal pathogen recognition by PPRs. During initial infection, fungal PAPMs are recognized by different host PPRs present in antigen-presenting cells (mainly Dendritic cells, macrophages, and neutrophils), interactions that trigger a downstream signal that induces the T cell naïve (CD4^+^ or CD8^+^) differentiation to Th17 cells (in the presence of TGFβ, IL-1, IL-6, and IL-23) and the subsequent production of IL-17 which in turn amplified the inflammatory response by recruiting neutrophils at the site of infection. However, each PRR plays a distinctly different role for each dimorphic fungus. Thus, *H. capsulatum* and *C. posadasii* are recognized by Dectin-1 and Dectin-2, *P. brasiliensis* is recognized by Dectin-1, TLR4, MR, and NLRP3, whereas *B. dermatitidis* is only recognized by Dectin-2. After recognition, the PRRs activate downstream adaptor molecules, including MyD88 and CARD9, that finally activate transcription factors that induce the specific genes, especially this coding for IL-17. Once the IL-17 is produced, it induces the recruitment and activation of neutrophils, which in turn exhibit microbicidal mechanisms mediated by antimicrobial peptides release, reactive oxygen species (ROS) production, and NETs formation. Of note, TLR2 and Gal3 negatively regulate IL-17 production after *P. brasiliensis* recognition. Moreover, the interaction of fungal cells (i.e. *Blastomyces*) with lung epithelial cells, induces the release of IL-1 which in turn activates innate cells that then induce activation and IL-17 production by innate cells, mainly nTh17 and γδTcells. PAMP, Pathogen-associated molecular pattern; PRR, pattern recognition receptor; Th, T helper; Tc, T cytotoxic; Dect, Dectin; MR, mannose receptor; TLR, Toll-Like receptor; Gal3, Galectin-3; NLRP3, NOD-like receptor P3; CARD9, adaptor recruitment domain family member 9; MyD88, myeloid differentiation factor 88; DCs, Dendritic cells; Mq, macrophages; PMN, polymorphonuclear neutrophils; IL, interleukin; TGFβ, transforming growth factor β; ROS, reactive oxygen species; NETs, extracellular traps; nTh17, natural T helper cells (innate cells).

In the dimorphic fungal infections, the induction of Th17 cells and the subsequent IL-17 production depends on the interactions of the PRRs present on the host cells’ surfaces. The fungal pathogen-associated molecular patterns (PAMPs), TLRs and C-Type Lectin [including Dectin-1, Dectin-2, MR and Mincle] receptors have been the most studied PPRs so far. These interactions induce the secretion of proinflammatory cytokines and T cell differentiation. **Figure 1** shows the interactions of the main dimorphic fungal pathogens and PRRs with the subsequent signaling activation (with adaptor molecules participation), Th17 differentiation, and IL-17 production.

### Histoplasmosis

Histoplasmosis is the most common endemic mycosis reported worldwide. The infection is acquired by the inhalation of aerosolized microconidia and mycelial fragments, but the severity of illness and the presence of clinical manifestations depend on the intensity of fungal burden exposure and the host´s immune status (Wheat et al., 2016). It is known that Th17 and its signature cytokine IL-17 play an important role and mediate a response in *H. capsulatum* infection; however, despite IL-17 not being necessary for survival, its neutralization alters inflammatory cell recruitment and elevates fungal burden in a murine model of histoplasmosis; therefore, it was demonstrated that this cytokine participates in the control of this fungal infection, particularly in the absence of IFN-γ (Deepe and Gibbons, 2009).

Among the different PRRs that participate in the recognition of *Histoplasma*, it has been reported that Dectin-1 and Dectin-2, but not Mincle, recognize and induce a protective Th17 immune response against *H. capsulatum* (Wüthrich et al., 2011; Viriyakosol et al., 2013; Wang et al., 2014); moreover, these interactions are mediated by Card9 and MyD88 signaling, which are indispensable for the development of this Th17 protective immune response (Wüthrich et al., 2011; Wang et al., 2014). Additionally, it has been demonstrated that the Galectin-3 (gal3) receptor, a member of the galectin family, negatively regulates IL-17A response through the inhibition of IL-23/IL-17 axis cytokine production by dendritic cells (DC) when infected with *H. capsulatum* (Wu et al., 2013). Similarly, it has been reported that the induction of IL-23 producing DCs depended on the activation of Dectin-1, which is mediated by β-glucan exposed in the cell walls of the fungal pathogens. Interestingly, the yeast form of *Histoplasma*, which lacks cell wall exposure of β-glucan, failed to induce IL-23 producing DCs, a fact that was confirmed using a mutant of *Histoplasma* in which β-glucan present in its cell wall was unmasked; thus, the interaction of DC with this mutant not only abrogated the pathogenicity of this fungus but also triggered the induction of IL-23 producing DCs (Chamilos et al., 2010); this study indicated that β-glucan exposure in the fungal cell wall is essential for the generation of IL-23 producing DCs and Th17 immune responses and may represent an evasion mechanism exerted by *Histoplasma*.

It is known that the differentiation of Th17 cells and their IL-17 production are mediated by IL-6 and TGF-β signaling, a process that is amplified and sustained by IL-21 and IL-23, respectively (Korn et al., 2009; Zhu et al., 2010). Along these lines, in *Histoplasma* infection, it has been described that the Th17 response is associated with cytokines that include IL-6, IL-23, and IL-17 and that CD4+ and CD8+ T cells expressing CD25 are the predominant sources of IL-17 (Deepe and Gibbons, 2009; Kroetz and Deepe, 2012). Moreover, the infection model for histoplasmosis showed that the development of the IL-17 protective response required IL-6 but not the participation of IL-1 receptor signaling (Nanjappa et al., 2012). More recently, it was reported that IL-22 deficiency was associated with a reduction of IFN-γ or IL-17-producing CD4+ cells in the lungs of mice infected with *H. capsulatum*, suggesting an inflammatory loop between IL-22 and a Th1/Th17 response (Prado et al., 2020).

In addition, some chemokines and their receptors also participate in the development of a Th17 response in *Histoplasma *infection; thus, the absence of CCR5 or CCL4 neutralization was associated with the impaired infiltration of the lungs of *Histoplasma-*infected mice by inflammatory cells. Those mice resolved the infection. The absence of CCR5 or CCL4 neutralization was also associated with the beneficial role of IL-17 accelerating the pathogen resolution (Kroetz and Deepe, 2010); moreover, mice lacking the CCR5 and treated with a monoclonal antibody against IL-17 showed an increase in the fungal burden and Treg cells, suggesting that this CCR5/CCL4 axis regulates the balance between Treg and Th17 cells in this fungal infection (Kroetz and Deepe, 2010). The important role of IL-17 in the *Histoplasma* infection has been clearly demonstrated, especially in immunization studies where this cytokine has been associated with the development of a protective immune response (Wüthrich et al., 2011; Deepe et al., 2018).

### Coccidioidomycosis

Coccidioidomycosis, commonly known as San Joaquin Valley fever, is a systemic fungal disease caused by the inhalation of the airborne spores of *Coccidioides immitis* or *C. posadasii* (Galgiani et al., 2005). The development of a protective immune response in *Coccidioides* infection is similar to that observed in histoplasmosis. Thus, Th1 and Th17 immune patterns are pivotal in mounting an effective control of coccidioidal infection (Hung et al., 2011; Wüthrich et al., 2011). Several studies employing different coccidioidal antigens and adjuvants for immunization protocols have been reported; these antigens include: a genetically-engineered mutant strain (Δcts2/ard1/cts3) used as a live attenuated vaccine (ΔT) (Xue et al., 2009); a ΔT conjugated with the adjuvant EP67; a peptide agonist of the biologically active C-terminal region of human complement component C5a (Hung et al., 2012); a multivalent recombinant *Coccidioides* polypeptide antigen (rCpa1) that consists of three previously identified antigens (Ag2/Pra, Cs-Ag, and Pmp1) and five pathogen-derived peptides with high affinity for human major histocompatibility complex class II (MHC-II) molecules (Hurtgen et al., 2012; Hung et al., 2018; Campuzano et al., 2020); and a rCpa1 encapsulated into glucan-chitin particles (GCP) or β-glucan particles (GP) (Campuzano et al., 2020). Additionally, transgenic mice expressing a human major histocompatibility complex class II (MHC II) receptor have also been employed to study the immune response to coccidioidal vaccines (Hurtgen et al., 2012; Hurtgen et al., 2016; Hung et al., 2018). All the above studies confirmed the protective effect addressed by Th1 and Th17 expansion with higher production of the signature cytokines, IFN-γ, and IL-17, respectively. Nonetheless, a higher expression of *RORc*, the hallmark transcription factor of the Th17 pathway combined with higher amounts of IL-23 and IL-6 and accompanied by a down-regulated expression of *Foxp3*, which promotes the differentiation of Treg cells, suggests the development of a biased Th17 protective immune response to coccidioidal infection (Hung et al., 2011; Wüthrich et al., 2011; Hung et al., 2012). Moreover, an additional study showed that IFN-γ^−/−^ knock-out mice immunized with the ΔT vaccine could still be protected (100% survival), while only 40% of ΔT-vaccinated IL17 receptor A-deficient mice survived (Hung et al., 2011); again, this study shows that Th17/IL-17 axis contributes strongly to protection against *Coccidioides* infection.

More recently, a primary dendritic cell (DC)-vaccine [DC-vaccine (Ag2-DC) that was prepared by non-virally transfecting the primary bone marrow-derived DCs with a plasmid DNA encoding Ag2/PRA (protective epitope of Coccidioides)] was evaluated, and healthy mice treated with the DC-vaccine showed IL-17, IFN-γ and IL-4 cytokine-secreting cells in the lungs and lymph nodes after immunization (Awasthi et al., 2019).

Regarding PRRs that participate in the recognition of *Coccidioides* and the subsequent stimulation and development of a protective Th17 immunity response, it has been demonstrated that this mechanism is mediated by the activation of both MyD88 and Card9-associated Dectin-1 and Dectin-2 signal pathways (Wüthrich et al., 2011; Viriyakosol et al., 2013; Wang et al., 2014; Campuzano et al., 2020). Moreover, it was also demonstrated that the IL-1 receptor, but not TLR2, is essential to developing a Th17 immunity response against *Coccidioides* infection, a mechanism mediated by MyD88 (Hung et al., 2016).

### Blastomycosis

Blastomycosis refers to a disease caused by the dimorphic fungi *Blastomyces dermatitidis*; this systemic disease, like endemic mycosis, has a T-cell and macrophage-mediated immune response (Chang et al., 2000). The clinical spectrum of this illness is broad, from asymptomatic patients to acute, chronic, or disseminated disease (Chapman et al., 2008). As described previously, in histoplasmosis and coccidioidomycosis, a Th1 and Th17 immune responses exert an important role in the control of infection by *Blastomyces* (Wüthrich et al., 2011; Wang et al., 2014). Thus, after the host cells recognize *Blastomyces´* fungal cell wall or its antigens by PRRs, activation and differentiation, mainly of Th17 cells, take place. Among the different PRRs that participate in *Blastomyces* recognition, Dectin-2 and Dectin-3 (also known as MCL) appear to be indispensable to developing a Th17 protective immune response and resistance to this fungal pathogen (Wüthrich et al., 2011; Wang et al., 2014; Wang et al., 2015; Wang et al., 2017). Of note, MCL regulates the development, expansion and, differentiation of Th17 through a mechanism dependent on the adaptor FcRγ (Wang et al., 2015). Furthermore, once MR recognizes a mannan-like structure on the *B. dermatitidis* cell wall, it also activates and differentiates naïve T cells into Th17 effector cells, which are pivotal to the protection of an immunized host against *Blastomyces* (Wang et al., 2016). Additional adaptor molecules, including Card9 and MyD88, are also indispensable for the development of a Th17 protective immune response against *Blastomyces* (Wüthrich et al., 2011; Wang et al., 2014; Nanjappa et al., 2015; Wang et al., 2017).

On the other hand, IL-1 and IL-6 appear also to play an important role in the development of a Th17 immune response against blastomycosis (Nanjappa et al., 2012; Wüthrich et al., 2013; Merkhofer et al., 2019). More recently, studies in animals and human beings have revealed that IL-6 had a pivotal role in the development of adaptive immunity and resistance to *B. dermatitidis* infection, through induction of a Th17 pattern. Thus, genetic analysis of an Asian population (The Hmong) that show an elevated incidence of blastomycosis in comparison with those of European ancestry (168 vs 13 per 100,000 inhabitants, respectively), demonstrated that in addition to mice that had lost IL-6 signaling, the presence of polymorphisms on the *IL-6* gene increased susceptibility to developing blastomycosis, a fact that was associated with lower levels of IL-6, IL-17, and RAR-related orphan receptor gamma t [(RORγt), the hallmark transcription factor of IL-17-producing T cells] in humans and low recruited Th17 producing cells in lungs of IL-6^−/−^ mice infected with *B. dermatitidis* (Merkhofer et al., 2019). By the same token, it has been reported that the use of exogenous IL-1 enhanced the protection of weak vaccines against lethal *B. dermatitidis* infection, promoting the development of fungus-specific Th17 cells (Wüthrich et al., 2013).

Recently, it has been described that lung epithelial cells are essential for immunity against *Blastomyces*; thus, the interaction of these epithelial cells with the fungus triggers a NF-κB signaling with a subsequent increase in the number of IL-17-producing innate lymphocytes, mainly CCR6^+^ natural Th17 cells (nTh17) and γδT-cells, a mechanism dependent on CCL20 chemokine production, which in turn is induced by IL-1α/IL1R signaling (Hernández-Santos et al., 2018).

Finally, vaccination against *Blastomyces* induces the development of memory Tc17 cells with different requirements for long-term persistence than Tc1 cells; thus, these anti-fungal Tc17 cells retained the expression of RORγt and showed higher proliferative renewal and lower levels of anti-apoptotic molecule Bcl-2, but required hypoxia-inducible factor 1α (HIF-1α) for their homeostasis (Nanjappa et al., 2017).

### Paracoccidioidomycosis

Paracoccidioidomycosis (PCM) is a fungal infection caused by the dimorphic fungus from the genus *Paracoccidioides* and is one of the most prevalent systemic mycoses in Latin America (Restrepo et al., 2019). In PCM, cellular immunity exhibits a protective role; thus, CD4^+^ T cells exert a protective effect through the regulation of antibody production and delayed-type hypersensitivity (DTH) reactivity, while CD8^+^ T cells control fungal burden (Chiarella et al., 2007). Furthermore, using a PCM model, it has been demonstrated that infected *P. brasiliensis-*infected mice showed increased levels of IL-17A accompanied by the Th17 associated cytokines, IL-6 and IL-23; moreover, deficiency of these Th17-associated cytokines or IL-17RA conferred susceptibility during infection associated with reduced concentrations of TNF-α, IFN-γ, and inducible nitric oxide synthase (iNOS) expression (Tristão et al., 2017).

Regarding participation of PRRs on Th17 development in PCM, it was demonstrated that TLR2 acts as a negative regulator of Th17 cells, thus a deficiency of TLR2 was associated with a lower fungal burden, a prevalent Th17 immunity (higher levels of IL-17, IL-6, IL-23, and TGF-b), and an increased number of neutrophils; nonetheless, a exacerbated pulmonary inflammation was also observed, which was associated with a diminished expansion of regulatory T cells (Loures et al., 2009). Similarly, TLR3 also acts as a negative regulator of Tc17 cells (IL-17-CD8^+^ producing cells); thus, in TLR3 deficient mice infected with *P. brasiliensis*, an increased number of Tc17 and Tc1 cells associated with higher levels of IL-17, IL-1β, IL-6, and IFN-γ were observed (Jannuzzi et al., 2019).

Conversely, *in vitro* studies showed that neutrophils primed with the 43kDa glycoprotein (gp43) from *P. brasiliensis* express higher levels of TLR2 and IL-17 (Gardizani et al., 2019). It is noteworthy that MyD88 (the adaptor molecule used by TLRs) is required to mount an efficient innate and adaptive immune response; thus, MyD88 deficient mice showed an association between disease severity and reduced Th17 response and IL-1β production (Loures et al., 2011).

Regarding CTL receptors, it has been reported that Dectin-1 has a critical influence in the differentiation and migration of Tc17 cells; thus, Dectin-1 deficient mice infected with *P. brasiliensis* showed a more severe infection, enhanced tissue pathology, and mortality rates, accompanied with reduced differentiation of T cells to Tc17 phenotype, increased expansion of Treg cells, impaired production of Th1, Th2, and Th17 cytokines, and migration of Tc17 and neutrophils to the site of infection (Loures et al., 2014). Moreover, monocytes from healthy individuals produced IL-17A after incubation with *P. brasiliensis* yeasts *via* activation of the Dectin-1 receptor (Romagnolo et al., 2018). Of note, dendritic cells stimulated with *P. brasiliensis* induced the differentiation of Th17/Tc17 by a mechanism mediated by TLR4, Dectin-1 and MR in a synergistic fashion (Loures et al., 2015).

Furthermore, activation of the NOD-like receptor P3 (NLRP3), which is related to the inflammasome, was associated with the development of protective immunity against *P. brasiliensis* by a mechanism mediated by Th1 and Th17 (Feriotti et al., 2017).

In another study using the experimental model of pulmonary paracoccidioidomycosis, it has been reported that IL-1α deficiency was associated with a reduction of Th17 cells and a diminished number of neutrophils in a mechanism mediated by caspase 11 (Ketelut-Carneiro et al., 2019). Moreover, the important role of IL-6 for the development of a protective Th17 immune response has been demonstrated; thus, the adoptive transfer of IL-6 competent macrophages restored the resistance in *P. brasiliensis*-infected IL-6- and IL-17RA-deficient mice (Tristão et al., 2017).

Interestingly, the results of other studies have been controversial; thus, we reported that during the early stages of infection, the depletion of neutrophils using a specific monoclonal antibody was associated with an exacerbation of the inflammatory response and high fungal burden. This neutrophil depletion was also accompanied by a decreased level of IL-17; moreover, it was confirmed that neutrophils were an essential source of IL-17 during the early stages of *P. brasiliensis* infection (Pino-Tamayo et al., 2016). Conversely, neutrophil depletion during the chronic course of paracoccidioidomycosis promotes the resolution of pulmonary inflammation and fibrosis accompanied by a reduced fungal burden. These results were associated with a decrease of proinflammatory cytokines, including IL-17, TNF-α, and TGF-β (Puerta-Arias et al., 2016). Additionally, the most severe form of PCM has been characterized by a predominant Th17/Th22 response, along with substantial participation of Th1 cells (de Castro et al., 2013). On these lines, it has been reported that *P. brasiliensis*-infected mice treated with a recombinant 60-kDa heat shock protein of this fungal pathogen increased the concentrations of proinflammatory cytokines, including IL-17, TNF-α, and IFN-γ, which lead to severe inflammation, host tissue damage, impaired granuloma formation, and fungal dissemination (Fernandes et al., 2016). The above results indicate that IL-17 plays a dual role in PCM.

Finally, in addition to CD4+Th cells, it has been demonstrated that other cell populations are important sources of IL-17, induced by dimorphic fungal pathogens; these cells include Tc17, nTh17, γδT-cells, and neutrophils (Pino-Tamayo et al., 2016; Nanjappa et al., 2017; Tristão et al., 2017; Hernández-Santos et al., 2018).

Altogether, the above findings suggest that the antifungal effect exerted by the Th17/IL-17 axis is mediated by the recruitment and activation of neutrophils and macrophages to the site of infection (Wüthrich et al., 2011; Nanjappa et al., 2012; Hernández-Santos et al., 2018).

## The Role of Neutrophils in The Dimorphic Fungal Infections

Neutrophils are the most abundant type of immune cells and constitute the first line of defense against infections by different pathogens. As multifunctional cells of the immune response, neutrophils actively participate in the development of innate and adaptative immunity (Kolaczkowska and Kubes, 2013), and exert a variety of effector functions including phagocytosis, intra and extra-cellular pathogen killing *via* oxidative and non-oxidative cytotoxic mechanisms, extracellular release of microbicidal molecules stored in their intracellular granules, production of immune mediators including pro-inflammatory cytokines and chemokines, and formation of neutrophil extracellular traps (NETs) (Mócsai, 2013; Wang and Arase, 2014; Tecchio and Casatella, 2016; Yang et al., 2017). The latter is one of the most important microbicidal mechanisms, mainly against certain pathogens that are difficult to phagocytose due to their large size (Brinkmann et al., 2004).

The microbicidal effect of NETs has been reported in different animal and human models with infections by fungal pathogens, parasites, bacteria, and viruses. This mechanism can comprise two phases: i) the trapping and immobilization of the pathogen to prevent the spread to tissues, organs, and systems; and ii) the elimination of the pathogen by microbicidal action of the proteins present in the NETs (Brinkmann et al., 2004). Such proteins exert their effect by degrading virulence factors, damaging the cell wall, or forming a complex with metal ions important in the life cycle of some dimorphic fungi (Brinkmann et al., 2004; Urban et al., 2006; Bianchi et al., 2009; Byrd et al., 2013; Hoeksema et al., 2016). However, although NETs can prevent the pathogens’ spread or directly eliminate the trapped microorganism, some studies have reported that certain peptides, as well as histones linked to the traps, trigger a high cytotoxic effect in the tissue, especially when the clearance mechanisms of the host are ineffective, thus causing a continuous inflammation as observed in several disorders or infectious diseases (Leffler et al., 2012; Cheng and Palaniyar, 2013).

Clearly, it is well known that the neutrophils are crucial in the immunity against invasive infection caused by fungal pathogens of the genus *Candida* and *Aspergillus* (Desai and Lionakis, 2018). However, in other fungal infections caused by a heterogeneous group of dimorphic fungi, including *Histoplasma capsulatum*, *Coccidioides* spp., *Paracoccidioides* spp., and *Blastomyces dermatitidis* (Hsu et al., 2010), this immune-cell apparently does not play an important role (Lionakis et al., 2017; Lionakis and Levitz, 2018). For this group of fungal infections, it has been suggested that these phagocytic cells may play a paradoxical function which depends on both the infection phase (acute or chronic) or certain conditions of the host; thus, neutrophils could exert a beneficial effect by controlling the infection, or, on the contrary, they could induce a detrimental effect with a poor prognosis of the infection/disease and worse outcomes. In **Table 1**, we summarized the role of neutrophils in the main dimorphic fungal infections.

**Table 1:** The role of neutrophils in dimorphic fungal infections.

Fungal infection	Findings	References
**Histoplasmosis**	• Fungal recognition is mediated by Dectin-2, NLRP3, and Mac-1 (CD11b/CD18) • Opsonized fungal cells are mediated by CR1, CR3, and FcRIII (CD16) • LTB4, LTC4, PAF, KC, and TNF-α induce neutrophil recruitment • Exhibited limited fungicidal activity • Azurophilic granules containing defensins and serprocidins exert a fungistatic effect • Fungus inhibit apoptosis and decreases Mac-1 expression • NETs formation exerts fungicidal activity *via* ROS, SyK/Src kinase, and CD18	• Coxon et al., 1990; Patin et al., 2019 • Newman et al., 1993 • Medeiros et al., 2015 • Kurita et al., 1991 • Brummer et al., 1991 • Newman et al., 2000 • Medeiros et al., 2002 • Thompson-Souza et al., 2020
**Coccidioidomycosis**	• Neutrophils are the predominant inflammatory cells located surrounding mature spherules • Spherules and endospores are recognized *via* TLR-2 and Dectin-1 • Source of IL-10 • NETs and granuloma formation prevent fungal dissemination • Genetic patterns and pre-exposition influence phagocytic functionality • Presence of NOX2 limits neutrophil recruitment in coccidioidal infection	• Galgiani et al., 2005; Shubitz et al., 2008; Xue et al., 2009. • Lee et al., 2015 • Hung et al., 2013 • Shubitz et al., 2008 • Hung et al., 2014 • Gonzalez et al., 2011
**Blastomycosis**	• Deleterious, enhance fungal replication and exacerbation of infection • Phagocytic cells exhibit capability to kill fungus • Pyogranuloma formation mediated by CXC chemokines • Neutrophil-DC hybrid exhibit a better fungicidal function, NETs formation, and ROS production	• Brummer and Stevens, 1983 • Brummer and Stevens, 1984; Brummer et al., 1986; Morrison et al., 1989 • Lorenzini et al., 2017 • Fites et al., 2018
**Paracoccidioidomycosis**	• Essential during early infection (protection) and source of IL-17 • Deleterious during chronic infection • Presence of Type I and Type II subsets • NETs formation and prevention of fungal dissemination • Fungal cells are recognized *via* Dectin-1, MR, TLR2, and TLR4 • Fungal stimulation induces IL-12, IL-10, PGE2, and LTB4 • IL-8 production inhibits apoptosis and allows fungal replication • Paracoccin induces: IL-8, IL-1β, ROS production, DNA release and inhibit apoptosis • Phagocytic cells primed with IFN-γ, IL-1β, GM-CSF, TNF-α, and IL-15 exhibit antifungal activity and trigger respiratory burst • Host genetic background influences their immunoregulatory functions	• Pino-Tamayo et al., 2016 • Puerta-Arias et al., 2016 • Puerta-Arias et al., 2018 • Mejía et al., 2015; Della-Coletta et al., 2015; Bachiega et al., 2016 • Acorci-Valério et al., 2010; Balderramas et al., 2014; Gardizani et al., 2019 • Balderramas et al., 2014 • Acorci et al., 2008 • Ricci-Azevedo et al., 2018 • Kurita et al., 2000; Tavian et al., 2007; Rodrigues et al., 2007 • Pina et al., 2006; Sperandio et al., 2015

### Histoplasmosis

Although the T-cell activated macrophages play a central role in the pathogenesis of histoplasmosis, it has also been suggested that *H. capsulatum* yeasts are recognized by different phagocytic cells, including neutrophils (Deepe et al., 2008). Earlier studies in murine models of histoplasmosis have described the presence of neutrophils during the first 36 h post-infection (Procknow et al., 1960; Baughman et al., 1986). Thus, recognition of *Histoplasma* yeast cells by neutrophils is mediated by PRRs present on their surface; these PRRs include Dectin-2, NLRP3, and macrophage integrin or integrin αMβ2 (Mac-1 or CD11b/CD18) (Coxon et al., 1990; Patin et al., 2019). Notably, phagocytosis of *H. capsulatum* yeast did not induce a respiratory burst response in neutrophils; however, the production of superoxide anion was observed only when fungal cells were opsonized, indicating that the activation of this microbicidal mechanism needs the participation of either complement or Fc receptors (Schnur and Newman, 1990). Later, Newman et al. (1993) demonstrated that recognition and phagocytosis of opsonized yeast of *H. capsulatum* by neutrophils was *via* complement receptor (CR) type 1 (CR1), CR3, and FcRIII (CD16).

Additionally, Medeiros et al. (2015) demonstrated that during the acute pulmonary histoplasmosis, inflammatory mediators such as leukotriene B4 (LTB4), LTC4, platelet-activating factor (PAF), KC (murine IL-8 homolog), and TNF-α induce a strong recruitment of neutrophils into the lungs and others remote localized inflammatory sites, where these cells exhibit fungistatic activity against *H. capsulatum*. Likewise, other reports have demonstrated the limited fungicidal effect of neutrophils against *H. capsulatum* (Brummer et al., 1991*;* Kurita et al., 1991). Newman et al. (2000) reported that the azurophilic granules present inside neutrophils contain defensins and serprocidins, molecules that exert a fungistatic effect against *H. capsulatum* yeast. It has also been demonstrated that *H. capsulatum* inhibits apoptosis in neutrophils from both human and mouse hosts, which correlates with decreased cell-surface Mac-1 expression (Medeiros et al., 2002). Overall, it could be suggested that *H. capsulatum* can evade microbicidal mechanisms or is able to survive inside the phagocytic cells during the early phase of infection.

Moreover, a recent report has shown that NETs response promotes the loss of yeast viability and exerts a fungicidal activity, as a dependent mechanism of ROS, SyK/Src Kinase pathway, and CD18 (Thompson-Souza et al., 2020).

Although it has been described that neutrophils participate in the innate and adaptive immune response, the role of these phagocytic cells in the cell-mediated immune response against *H. capsulatum* at early times of infection is still unclear.

### Coccidioidomycosis

The immunity against coccidioidomycosis is mediated mainly by macrophages and T cells, and relatively little is known about the role of neutrophils in the inflammatory response. Although the capacity of mononuclear cells to locate around parasitic-phase structures of the fungi has been shown, some studies have suggested that neutrophils may participate during the early course of the disease; thus histopathological examinations of the infected lungs of mice at the first two weeks postchallenge have shown that neutrophils are the predominant inflammatory cells located adjacent to mature spherules that have ruptured and released their endospores (Galgiani et al., 2005; Shubitz et al., 2008; Xue et al., 2009). It has been suggested that these phagocytic cells respond to the contents of spherules in a chemotaxis-like fashion, and the intense inflammatory response observed at infection sites may contribute to lung tissue damage, which could exacerbate the course of the disease (Hung et al., 2005). Moreover, it has been hypothesized that neutrophils could recognize the spherules and endospores of *Coccidioides* spp. *via* TLR2 or C-type lectin receptors, including Dectin-1, and inhibit their growth through NETs or granuloma formation. In the latter structure, the neutrophils are organized to form a necrotic center accompanied by eosinophilic debris and macrophages (Shubitz et al., 2008; Gonzalez et al., 2011; Lee et al., 2015). However, it is important to mention that in a mouse model of coccidioidomycosis, it was observed that lung-infiltrated neutrophils produce high amounts of IL-10, a fact that was associated with impairment of resistance to coccidioidal infection due to a suppression of Th1, Th2, and Th17 immunity mediated by this anti-inflammatory cytokine (Hung et al., 2013).

Paradoxically, Hung et al. (2014) observed that neutrophil-depleted mice infected with spores of the virulent isolate of *C. posadasii* did not show a difference in the fungal burden or the survival rate in comparison with control mice, indicating that neutrophils are dispensable for defense against this mycosis. Nonetheless, when the mice were immunized with a live-attenuated vaccine against coccidioidomycosis, the vaccine-induced protection promoted early recruitment and elevated numbers of neutrophils to the infection site, suggesting that the role of these phagocytic cells depends on prior exposure of the host to *Coccidioides* spp. (Hung et al., 2014).

In additional studies using mice deficient of NADPH oxidase 2 (NOX2), it was reported that NOX2 deficient mice infected with *Coccidioides* showed a reduced survival accompanied by a high and sustained number of lung-infiltrated neutrophils on days 7 and 11 postchallenge compared to infected WT mice. This evidence suggests that NOX2 production plays a role in limiting neutrophil recruitment and the subsequent pathogenic inflammation in this murine model of coccidioidomycosis (Gonzalez et al., 2011).

### Blastomycosis

In contrast with the other dimorphic fungal infections, immunosuppression like HIV/aids does not appear to be a risk factor in developing blastomycosis (Pappas et al., 1993; Schwartz and Kauffman, 2020); thus, neutrophils and other innate immune cells might be enough to control the infection.

Early *in vitro* studies with murine neutrophils have been controversial. Although the ability of neutrophils to kill *B. dermatitidis* has been demonstrated (Brummer and Stevens, 1984; Brummer et al., 1986; Morrison et al., 1989), some reports have shown that neutrophils enhance and allow the replication of this fungal pathogen, a fact that was associated with an exacerbation of the infection by accumulation and death of neutrophils in the tissue lesions (Brummer and Stevens, 1983). In this sense, the presence of a fungal chemotactic factor in serum-free culture filtrated of *B. dermatitidis* has been demonstrated (Sixbey et al., 1979; Thurmond and Mitchell, 1984). Lorenzini et al. (2017) also demonstrated that *Blastomyces* produces a peptidase (DppIVA) that cleaves chemokines, specifically those belonging to the CXC family, including the CXCL-2, which is the most potent chemoattractant molecule for neutrophils, thus improving neutrophil migration and promoting a pyogranulomatous response, a typical reaction during blastomycosis infection.

The function of a subset of neutrophils has recently been described in a murine model of pulmonary blastomycosis, which shows the capability of transdifferentiation in a neutrophil-dendritic cell hybrid that was associated with a better fungicidal function, NETs formation, and a higher expression of PRRs and the production of reactive oxygen species than canonical neutrophils (Fites et al., 2018). Although the role of these cells in other fungal infections is still unknown, these cells could be expected to contribute significantly due to their ability to improve both the innate and the adaptative immunity.

### Paracoccidioidomycosis

In contrast to other endemic mycoses, several studies have suggested the dual role played by neutrophils during PCM infection. The functionality of these phagocytic cells appears to depend on some factors, including the genetic pattern of the host or the stage of infection. Pina et al. (2006) observed a significant difference, in the role of neutrophils, between resistant and susceptible mice; thus, in susceptible mice, these phagocytic cells have low fungicidal activity, but in contrast, neutrophils from resistant mice are more abundant in the lesion areas and efficient to control infection. Similar results were obtained by Sperandio et al. (2015), who using resistant and susceptible mice to PCM showed that in susceptible mice, the infection was able to disseminate to their bone marrow, impairing the production and maturation of neutrophils, which is different from what was observed in resistant mice.

Similarly, it has been suggested that neutrophils are essential during the acute inflammatory phase since they represent more than 85% of inflammatory cells and could positively modulate the innate immune response through the production of pro-inflammatory cytokines and lipidic mediators in the infected lung tissue (Gonzalez and Cano, 2001; Gonzalez et al., 2003; Balderramas et al., 2014). Along these lines, using an experimental model of pulmonary PCM in mice with intermediate susceptibility to infection and treated with a monoclonal antibody specific to neutrophils, we reported that compared to control mice, infected and neutrophil-depleted mice showed decreased survival rates during the early stage of infection, accompanied by an increase in both the fungal burden and the inflammatory response with an exaggerated production of several chemokines and proinflammatory cytokines, suggesting the pivotal role of these phagocytic cells in this fungal infection during the early course of infection (Pino-Tamayo et al., 2016).

Conversely, in studies of chronic pulmonary PCM in those mice with intermediate susceptibility, it was reported that treatment with the antifungal itraconazole or the immunomodulator pentoxifylline, alone or in combination, was associated with a decreased number of neutrophils as well as with an improved outcome of the disease, suggesting that these phagocytic cells appear to play a deleterious effect during the chronic stages of PCM (Naranjo et al., 2010., Naranjo et al., 2011; Lopera et al., 2015). Subsequently, we reported that those mice treated with the monoclonal antibodies specific to neutrophils during the chronic stages of infection showed better control of infection correlated with a reduction not only on the fungal burden but also on the inflammatory response and pulmonary fibrosis, suggesting that these phagocytic cells appear to play a detrimental effect during the chronic course of pulmonary PCM (Puerta-Arias et al., 2016).

Additional studies confirmed the presence of two different subsets of murine neutrophils, the type I neutrophils associated with a pro-inflammatory response, and the type II neutrophils associated with an anti-inflammatory response. Thus, a greater number of type II neutrophils were observed, which could be related to the incapacity of the host to control *P. brasiliensis* infection without the appropriate treatment (Puerta-Arias et al., 2018).

Moreover, other reports have described the capacity of *P. brasiliensis* to survive inside neutrophils and extend the lifetime of these cells *via* IL-8 production triggered by the fungus, suggesting that *P. brasiliensis* could evade the antifungal mechanisms allowing its replication (Brummer et al., 1989; Acorci et al., 2008). Along the same lines, it has been reported that paracoccin, a lectin expressed by *P. brasiliensis*, induces IL-8, IL-1β, and ROS production. It also induces the release of DNA and inhibits apoptosis (Ricci-Azevedo et al., 2018). In contrast, it has been reported that neutrophils exhibit antifungal activity against *P. brasiliensis* yeast only when these phagocytic cells are priming with IFN-γ, GM-CSF, IL-1β, and TNF-α (Kurita et al., 2000; Rodrigues et al., 2007). Similar results were obtained when human neutrophils were activated with IL-15, which increases *P. brasiliensis* killing by a mechanism dependent on H_2_O_2_ and superoxide anion (Tavian et al., 2007).

On the other hand, other studies have reported that neutrophils recognize *P. brasiliensis* yeasts *via* Dectin-1, MR, TLR2, and TLR4 (Acorci-Valéro et al., 2010; Balderramas et al., 2014; Gardizani et al., 2019) and produce cytokines including IL-12, IL-10, PGE2, and LTB4 (Balderramas et al., 2014). Additionally, these interactions were able to induce NETs formation by either dependent or independent reactive oxygen species production, correlating with the fungal morphotype used for stimulation (conidia or yeast, respectively); however, the killing of the fungus by NETs was dependent on the fungal strain and previous activation by cytokines, including TNF-α, IFN-γ, and GM-CSF. In addition, NETs appear to prevent fungal dissemination (Mejía et al., 2015; Bachiega et al., 2016). Likewise, histopathological samples of cutaneous lesions from human PCM patients have revealed the production of NETs (Della-Coletta et al., 2015).

Taken all together, the above studies clearly demonstrated the important effector and immunomodulatory roles played by neutrophils during the early stages of infection, contributing to *P. brasiliensis* host resistance. Paradoxically, these phagocytic cells could also play a detrimental role during the chronic or advanced stages of infection.

## Neutrophils and IL-17 Participation in The Granulomatous Inflammation and Pulmonary Fibrosis Development of Endemic Mycoses

The lungs are the primary organs affected by the dimorphic fungal pathogens; thus, once the conidia reach the alveoli, they transform into pathogenic morphotypes (yeast cells or spherules). Initially, these fungal propagules interact with lung epithelial cells or alveolar macrophages. Such interactions trigger the activation of these host cells, which, in turn, secret soluble mediator molecules, mainly chemokines and pro-inflammatory cytokines that induce the recruitment of pro-inflammatory cells, including neutrophils and other innate cells, into the lungs (Martin and Frever, 2005). During pulmonary inflammation, neutrophils can also interact with epithelial cells, lymphocytes, macrophages, and other granulocytes inducing the activation of the adaptive immune response (Siew et al., 2017). However, neutrophils may be self-defeating; thus, they could play a protective role but could also contribute to injury and tissue damage, especially in those cases of chronic inflammation due to a continuous activation mediated by the IL-17 (Parkos, 2016; Rosales et al., 2016). Neutrophils contain a great number of proteases, inflammatory mediators, and oxidants stored in their granules that lead to the progression of pulmonary complications such as asthma, chronic obstructive pulmonary disease (COPD), granulomatous lesions, and finally fibrosis (Liu et al., 2017).

In the case of systemic and endemic mycoses, there are scant studies that link the IL-17 and/or neutrophils with the development of a chronic inflammatory process or with fibrosis development. Some studies have demonstrated the presence of neutrophils in pulmonary infiltrating cells during infections by *P. brasiliensis* (Gonzalez and Cano, 2001; Gonzalez et al., 2003), *H. capsulatum* (Baughman et al., 1986), *C. posadasii* (Hung et al., 2005), and *B. dermatitidis* (Lorenzini et al., 2017). However, as mentioned above, the role of neutrophils during these dimorphic fungal infections is not clear; moreover, their participation in the immune response appears to depend on the phase of infection (acute or chronic) (Pino-Tamayo et al., 2016; Puerta-Arias et al., 2016). Only in an experimental model of paracoccidioidomycosis, the role of neutrophils in the pulmonary fibrosis development has been studied, where it was demonstrated that these phagocytic cells are detrimental and promote granulomatous inflammation and pulmonary fibrosis during the chronic course of the mycosis, a process that was also associated with the presence of IL-17 (Pino-Tamayo et al., 2016; Puerta-Arias et al., 2016). Moreover, it was demonstrated that the Th17-associated cytokines, IL-17, IL-6, and IL-23, are crucial for granuloma formation during experimental paracoccidioidomycosis; thus, deficiency of IL-6, IL-23, or IL-17RA impaired the compact granuloma formation and conferred susceptibility to infection (Tristão et al., 2017). In an additional study conducted by Heninger et al. (2006), it was reported that the granuloma lesions induced by *Histoplasma* infection are composed mainly of CD4^+^, CD8^+^, Dendritic cells, and macrophages, which are the principal sources of IFN-γ and IL-17; notably, neutrophils were not evidenced. In the other fungal endemic and systemic mycosis (coccidioidomycosis and blastomycosis), the role played by neutrophils and IL-17 in the development of the fibrosis process remains to be explored.

On the other hand, pulmonary fibrosis (PF) is the final result of a chronic inflammation caused by microbial pathogens and chemical or physical agents; thus, PF is a consequence of a repetitive process of injury and reparation of the alveolar epithelium, which leads to an exacerbated wound healing process, accompanied by an excess deposition of extracellular matrix (ECM) components and a scarring process of the lung (Rydell-Tormanen et al., 2012; Chioma and Drake, 2017). Additionally, during this PF process, an increased number of myofibroblasts and fibroblasts that are known to synthesize connective tissue proteins have been observed, mainly Type III Collagen, and matrix metalloproteinases (MMP) (Baum and Duffy, 2011; Wynn and Ramalingam, 2012). In paracoccidioidomycosis, the presence of neutrophil infiltration within the granuloma is observed during the chronic form, and especially surrounding the granulomas (González et al., 2008). Additional studies have shown that treatment with a combination of Itraconazole plus an immunomodulatory agent (Pentoxifylline) during the chronic pulmonary paracoccidioidomycosis, reduced the granulomatous inflammation and neutrophils (Naranjo et al., 2011). Along the same lines, in this fungal model and using a monoclonal antibody specific to neutrophils it was demonstrated that the depletion of these phagocytic cells was associated with an attenuation of the inflammation and the fibrotic process through a down-regulation of pro-fibrotic mediators including IL-17, TGF-β1, TNF-α, MMP-8 and the tissue inhibitor of metalloproteinases (TIMP)-2 (Puerta-Arias et al., 2016). The above studies clearly suggest that both neutrophils and IL-17 are responsible, in part, for the fibrosis development, evidence that supports the idea that neutrophils and/or IL-17 can be targets of a therapeutic intervention for the treatment of fibrosis as well as mycosis.

## Future Questions and Conclusions

Over the last years, it has been learned that IL17 plays an important role and appears to be indispensable in developing a protective immunity against fungal infections including systemic and endemic mycoses, a mechanism that is mediated by the interactions between fungal PAMPs and PRRs with the subsequent activation and recruitment of neutrophils and macrophages at the site of infection. Furthermore, it has also been demonstrated that each PRR plays a distinctly different role for each dimorphic fungal pathogen. Nonetheless, both the IL17 and neutrophils play a detrimental role in inducing pathological disease, possibly due to an unrestrained inflammatory response. Future studies will need to focus on which specific fungal antigens or PAMPs are recognized by the different PRRs and confer immunity through this Th17 immune pattern or, on the contrary, which kind of interactions induce pathological disease. Additionally, it would be interesting to know if IL17 induce NETs formation as a microbicidal mechanism against these fungal dimorphic pathogens. Thus, understanding the specific interactions between fungal PAMPs and PRRs and their contributions to disease outcomes will provide potential insights for designing and developing immunotherapies to control these systemic and endemic mycoses.

## Author Contributions

All authors contributed to the article and approved the submitted version.

## Funding

Supported in part by Universidad de Antioquia; Basic and Applied Microbiology Research Group (MICROBA), School of Microbiology, Universidad de Antioquia, Medellin, Colombia.

## Conflict of Interest

The authors declare that the research was conducted in the absence of any commercial or financial relationships that could be construed as a potential conflict of interest.
